# Therapeutic perspective for children and young adults living with thalassemia and sickle cell disease

**DOI:** 10.1007/s00431-023-04900-w

**Published:** 2023-03-31

**Authors:** Marta Ferraresi, Daniele Lello Panzieri, Simona Leoni, Maria Domenica Cappellini, Antonis Kattamis, Irene Motta

**Affiliations:** 1 Unit of Medicine and Metabolic Disease, Fondazione IRCCS Ca’ Granda Ospedale Maggiore Policlinico, Università degli Studi di Milano, via F. Sforza, 35, 20122 Milan, Italy; 2 Università Degli Studi Di Milano, Milan, Italy; 3 Department of Clinical Sciences and Community Health, Università Degli Studi Di Milano, Milan, Italy; 4 Division of Pediatric Hematology-Oncology, First Department of Pediatrics, National and Kapodistrian University of Athens, Athens, Greece

**Keywords:** Thalassemia, Sickle cell disease, Luspatercept, Crizanlizumab, Gene therapy, Gene editing

## Abstract

Hemoglobinopathies, including thalassemias and sickle cell disease, are the most common monogenic diseases worldwide, with estimated annual births of more than 330,000 affected infants. Hemoglobin disorders account for about 3.4% of deaths in children under 5 years of age. The distribution of these diseases is historically linked to current or previously malaria-endemic regions; however, immigration has led to a worldwide distribution of these diseases, making them a global health problem. During the last decade, new treatment approaches and novel therapies have been proposed, some of which have the potential to change the natural history of these disorders. Indeed, the first erythroid maturation agent, luspatercept, and gene therapy have been approved for beta-thalassemia adult patients. For sickle cell disease, molecules targeting vaso-occlusion and hemoglobin S polymerization include crizanlizumab, which has been approved for patients ≥ 16 years, voxelotor approved for patients ≥ 12 years, and L-glutamine for patients older than 5 years.

*Conclusion:* We herein present the most recent advances and future perspectives in thalassemia and sickle cell disease treatment, including new drugs, gene therapy, and gene editing, and the current clinical trial status in the pediatric populations.

**Table:** 

**What is Known:** *• Red blood cell transfusions, iron chelation therapy and hematopoietic stem cell transplantation have been the mainstay of treatment of thalassemia patients for decades.* *• For sickle cell disease, until 2005, treatment strategies were mostly the same as those for thalassemia, with the option of simple transfusion or exchange transfusion. In 2007, hydroxyurea was approved for patients ≥ 2 years old.*	
**What is New:** *• In 2019, gene therapy with betibeglogene autotemcel (LentiGlobin BB305) was approved for TDT patients ≥ 12 years old non β0/β0 without matched sibling donor.* *• Starting from 2017 several new drugs, such as L-glutamine (approved only by FDA), crizanlizumab (approved by FDA and EMA for patients ≥ 16 years), and lastly voxelotor (approved by FDA and EMA for patients ≥ 12 years old).*

**What is Known:**

*• Red blood cell transfusions, iron chelation therapy and hematopoietic stem cell transplantation have been the mainstay of treatment of thalassemia patients for decades.*

*• For sickle cell disease, until 2005, treatment strategies were mostly the same as those for thalassemia, with the option of simple transfusion or exchange transfusion. In 2007, hydroxyurea was approved for patients ≥ 2 years old.*

**What is New:**

*• In 2019, gene therapy with betibeglogene autotemcel (LentiGlobin BB305) was approved for TDT patients ≥ 12 years old non β0/β0 without matched sibling donor.*

*• Starting from 2017 several new drugs, such as L-glutamine (approved only by FDA), crizanlizumab (approved by FDA and EMA for patients ≥ 16 years), and lastly voxelotor (approved by FDA and EMA for patients ≥ 12 years old).*

## Introduction

**Fig. 1:**
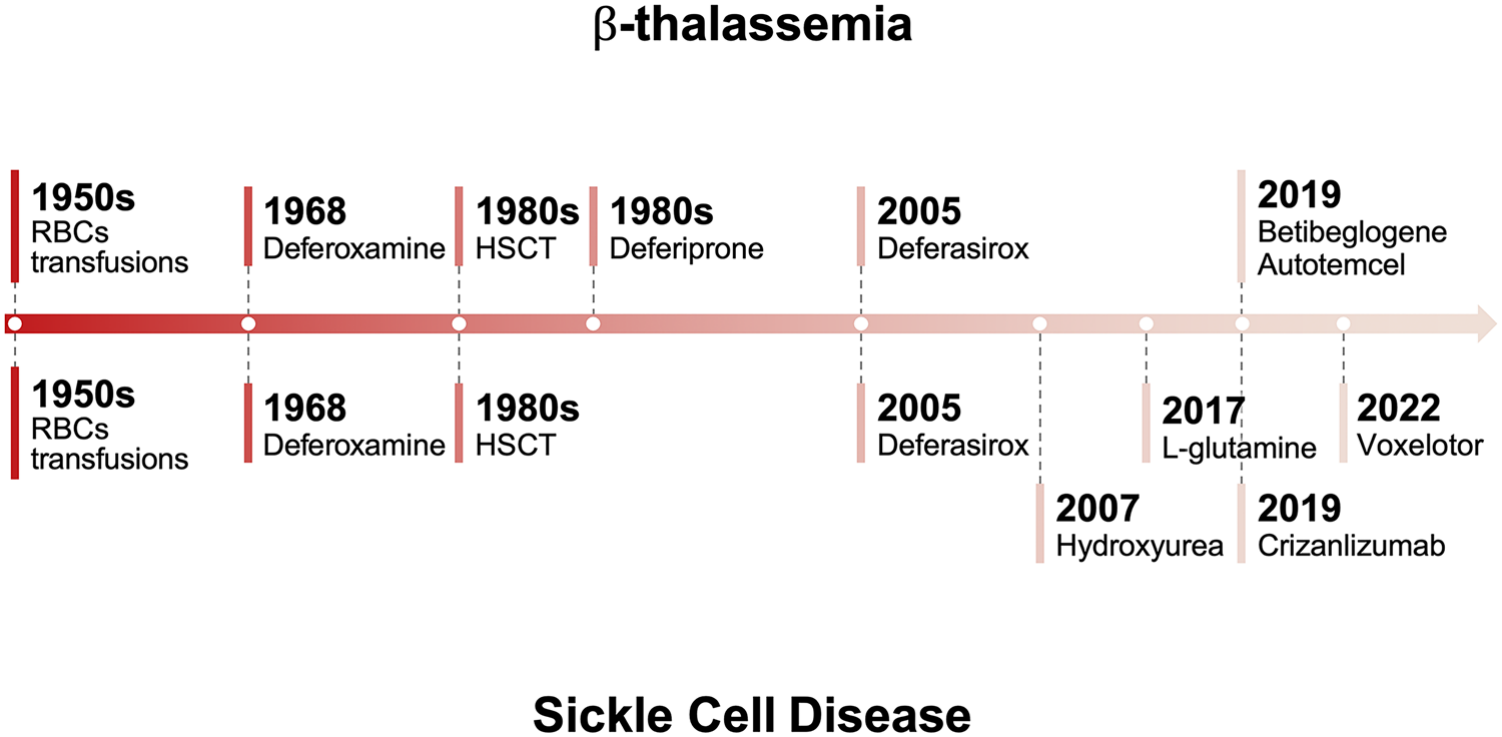
Milestones in the development of treatments for thalassemias and sickle cell disease. RBC, red blood cell; HSCT: hematopoietic stem cell transplantation

Hemoglobinopathies, including thalassemias and sickle cell disease (SCD), are the most common monogenic diseases worldwide, with a WHO report of 2008 estimating an annual birth of over 330,000 affected infants (83% SCD, 17% thalassemias). Hemoglobin (Hb) disorders account for about 3.4% of deaths in children less than 5 years of age [1]. However, there are several areas where the burden of these diseases is significantly higher, as for SCD in Tanzania and Nigeria, where mortality for children under 5 years old reaches 6–10% [2, 3]. The distribution of these diseases is historically linked to malaria since the red blood cell (RBC) abnormalities protect against the parasite infection. Therefore, the prevalence of SCD and thalassemia is higher in Africa, the Mediterranean Sea, the Middle East, India, and Southeast Asia [4, 5]. Immigration patterns have led to a worldwide distribution of these diseases, making them a global health problem [6–8]. These disorders are still often underdiagnosed, and disease-modifying therapies have been limited for many years. During the last decade, new treatment approaches and novel therapies have been proposed, some of which have the potential to change the natural history of these disorders (Fig. 1) [9].

## Thalassemias

Thalassemias are autosomal recessive disorders with reduced production of α- or β-globin chains [7] leading to unbalanced globin chain ratio and consequently to ineffective erythropoiesis, increased hemolysis, and altered iron homeostasis [10, 11]. During the last decades, attention has been mainly focused on β-thalassemia since it is the more clinically relevant form, and its most severe phenotype, previously named β-thalassemia major, allows postpartum survival, although with reduced life expectancy and quality of life [12]. On the contrary, the most severe form of α-thalassemia, namely Hb Bart’s, was considered fatal for the fetus until recently. In fact, fetal therapy with intrauterine blood transfusions during gestation is now feasible, allowing the correction of anemia, fetal growth, and prevention of hydrops; it also decreases preterm deliveries and contributes to infants having higher Apgar scores [13–15]. At present, thalassemia syndromes are classified as non-transfusion-dependent thalassemia (NTDT) and transfusion-dependent thalassemia (TDT) according to their clinical features and blood transfusions requirement [16, 17]. Conventional management is comparable in children and adults, and for TDT patients, it primarily relies on transfusion and iron chelation therapy, as well as splenectomy in selected cases [17]. As far as NTDT patients, the only currently approved therapy is iron chelation (for patients ≥ 10 years old) since there is evidence of clinically significant iron overload and subsequent multiorgan morbidity even in NTDT patients who never received transfusion therapy [18]. Transfusion programs and iron chelation therapies have been highly optimized, leading to increased survival in TDT patients born from 1985 [19]. However, these strategies still hold many challenges and limitations, and patients and physicians look forward to advances in novel therapies that can further decrease the burden of the disease [16].

Emerging therapies for β-thalassemia can be divided into three categories based on their pathological target: addressing ineffective erythropoiesis and/or hemolysis, modifying iron metabolism, and altering globin gene expression [16, 20]. Data on new therapies regarding children are currently limited as most clinical trials involve only adults at first.

As far as targeting ineffective erythropoiesis, luspatercept is a novel recombinant fusion protein that binds ligands of the transforming growth factor beta (TGF-β) superfamily, thus inhibiting SMAD2/3 signaling and therefore promoting late-stage erythropoiesis [21, 22]. The results of the phase III BELIEVE trial [23] led to the approval of luspatercept for the treatment of anemia in adult patients with TDT by the Food and Drug Administration (FDA) [24] and European Medicines Agency (EMA) [25, 26]. A phase II study to evaluate the safety and pharmacokinetics of luspatercept in TDT young patients ≥ 6 to < 18 years old is ongoing (Table 1). The study is divided into 2 parts: part A in participants aged 12 to 18 years with two dose escalation cohorts of 6 participants each, followed by a dose expansion cohort of 30 participants. Part B will begin after a review of the safety of participants completing at least 1 year of treatment in part A and will be in participants aged 6 to < 12 with two dose escalation cohorts of 6 participants each. Recently, a phase II trial on luspatercept encompassing only the NTDT adult population met the primary endpoint of achieving an increase from baseline of ≥ 1 g/dL in Hb levels in the 12 weeks between weeks 13 and 24 after the beginning of the treatment with luspatercept without transfusional support [27].

**Table 1:** Ongoing trial on novel pharmacological treatment under investigation in children with thalassemias

**Drug**	**Trial name/NCT**	**Population**	**Target**	**Phase/status**
**Luspatercept**	NCT04143724	β-TDT patients aged 6 to 18 years old	Ineffective erythropoiesis	Phase 2, recruiting
**PTG-300**	NCT03802201	β-TDT and NTDT patients aged 12 to 65 years old	Iron metabolism	Phase 2, terminated early for lack of efficacy
**Vemifeport (VIT-2763)**	NCT04364269 (VITHAL)	β-NTDT patients aged 12 to 65 years old	Ineffective erythropoiesis	Phase 2a, completed
**In utero HSCT**	NCT02986698	Fetuses with α-TDT	Genetic defect	Phase 1, active not recruiting

Small allosteric activators of the erythrocyte pyruvate kinase (PKR), which increase adenosine triphosphate (ATP) production, thus reducing levels of 2,3-diphosphoglycerate (2,3-DPG), have shown promising initial data for the treatment of hemoglobinopathies [28, 29]. The first in-class molecule, mitapivat (AG438), has been recently approved for the treatment of pyruvate kinase deficiency [30]. Studies on β-thalassemia mouse model showed that mitapivat ameliorates ineffective erythropoiesis, anemia, and iron overload [31]. In a phase II trial on NTDT adults with α- and β-thalassemia, it led to an Hb levels’ increase and improvement of hemolytic and erythropoietic markers with a good safety profile [29, 32]. Two phase III trials are currently underway with the primary objective to compare its effect on anemia and on transfusion burden in TDT and NTDT adults with α- or β-thalassemia [33, 34]. Another molecule evaluated for both β-thalassemia and SCD is IMR-687, a phosphodiesterase 9 (PDE-9) inhibitor that increases cyclic guanosine monophosphate (cGMP) levels and has shown in preclinical studies to increase fetal hemoglobin (HbF). The phase II trial on β-thalassemia showed a good tolerability profile of the drug, but it failed to show meaningful benefit in transfusion burden or improvement of hemolytic markers; thus, it was discontinued [35].

Molecules targeting iron metabolism are being studied in patients with thalassemia. Vamifeport (VIT-2763) is a small oral molecule that acts as ferroportin inhibitor. The results of the preclinical and phase I studies have led to a phase IIa double-blind, randomized, placebo-controlled study with the primary endpoint of assessing the safety and tolerability of vamifeport compared to placebo in NTDT patients ≥ 12 years. The trial has been completed in November 2021, but the results are yet to be published. A phase IIb trial including adults with TDT is being planned with the primary objective to evaluate the efficacy of several increasing doses of vamifeport as measured by reduction in RBC transfusion burden [36]. Other molecules with hepcidin-like action may reduce iron absorption and redistribution and improve erythropoiesis. However, so far, studies on two of these molecules showed lack of efficacy and were terminated early [37, 38]. Apotransferrin is another molecule targeting iron dysregulation. It upregulates hepcidin and downregulates transferrin receptor 1 [39], reducing iron absorption from the gut and potentially cardiac iron loading [40]. Apotransferrin is being evaluated in a phase II trial involving NTDT patients ≥ 17 years old [41]. Upregulating hepatic hepcidin production by manipulating transmembrane serine protease 6 (TMPRSS6) expression has shown promising results in β-thalassemia mouse models by reducing iron burden and improving ineffective erythropoiesis. Clinical trials on the use of NTDT patients with two different agents are currently ongoing [42–44].

## Sickle cell disease

Sickle cell disease is characterized by the presence of hemoglobin S (HbS), which polymerizes when deoxygenated, damaging the erythrocytes and causing vaso-occlusive crises (VOCs) and chronic hemolytic anemia [4]. VOCs are acute vaso-occlusive events causing ischemia in several organs and severe acute pain. They are complex events still only partially understood, involving the interactions between dense RBC, reticulocytes, abnormally activated endothelial cells, leukocytes, platelets, and plasma factors [45], leading to vasculopathy, altered adhesion, inflammation, and activation of coagulation pathways [46]. SCD is a multisystem disease associated with organ damage due to acute events (VOCs) and chronic complications, such as cerebrovascular disease, pulmonary hypertension, hyposplenism, and renal failure, leading to shortened lifespan and decreased quality of life [4]. While children’s mortality is mostly due to acute events, adults’ mortality is more related to chronic complications [47]. Until recently, clinical interventions were based mainly on supportive care therapies, such as interventions to reduce infection risk, like prophylactic antibiotics and vaccinations against capsulated bacteria. Hydroxyurea, bone marrow transplantation, and chronic transfusion therapy for stroke prevention have been the only available modifying treatments for SCD. However, the therapeutic intent, efficacy in preventing the progression of the disease, adverse effects, costs, and patient burden vary greatly between these therapies [48]. In particular, hydroxyurea has been shown to prevent chronic and acute complications of SCD in both children and adults [49]. It increases HbF levels, thereby decreasing HbS concentrations and its polymerization. Additional mechanisms of action include reduction of the neutrophil and platelet count, which improves the altered cell adhesion-inflammation pathways, and correction of the nitric oxide (NO) deficiency state caused by chronic hemolysis [49]. The drug is approved by FDA [50] and EMA [51] for SCD patients ≥ 2 years old for the prevention of recurrent painful VOC crises, including acute chest syndrome [52, 53]. Emerging evidence suggests that initiating hydroxyurea in the first year of life may have a neuroprotective effect on children with SCD, allowing a healthier adulthood [54]. Although hydroxyurea can prevent painful crises and is suggested to increase life expectancy [55, 56], a poor adherence to this therapy is still reported, mainly due to fears about the potential side effects [57]. The recent ESCORT-HU study demonstrated a clear clinical benefit of the therapy with hydroxyurea, along with good tolerance, despite infrequent clinical and biological follow-up, supporting its use also in low-income countries. However, hydroxyurea is not a cure, and the recurrence of VOC events may still occur [58]. Adding new emerging therapies, alone or in combination with hydroxyurea, offers hope of reducing the remaining painful episodes. Current and future therapies target different pathophysiological mechanisms of SCD: (a) modulation of Hb polymerization, erythrocyte dehydration, and Hb oxygen affinity; (b) prevention of vaso-occlusion by inhibiting cells interactions; (c) prevention of endothelial dysfunction; and (d) modulation of inflammation [46]. Three novel drugs have been approved over the last few years for SCD treatment. The first one is L-glutamine, an amino acid used by the enzyme NAD + synthetase to produce NAD + from NADH, an essential cofactor in redox reactions whose requirement is increased in SCD. A phase 3 trial [59] showed a reduction in VOCs and hospital visits in both children and adults treated with L-glutamine, leading to its approval by FDA for patients older than 5 years [60]. The company withdrew the application for approval from EMA, after the agency had expressed concerns about the efficacy data. More recently, two other drugs, voxelotor and crizanlizumab, have been approved by FDA and EMA. The former is a Hb modulator that inhibits the polymerization of HbS stabilizing the hemoglobin in the oxygenated status. In the phase III clinical trial, voxelotor was effective in increasing hemoglobin and reducing hemolysis indices, and a tendency in VOCs reduction was observed [61]. It is approved for SCD patients ≥ 12 years old with persistent anemia and hemolysis despite hydroxyurea therapy titrated to the maximum tolerated dose or in those who are considered hydroxyurea-intolerant [62, 63]. Several studies assessing voxelotor in children are underway. A phase IIa trial assessing its pharmacokinetics, safety, tolerability, and efficacy in patients from 6 month to 17 years old is ongoing. The effects of voxelotor on the transcranial doppler ultrasound measurements in SCD patients ≥ 2 to < 15 years old are being studied in a phase III trial. Lastly, a phase III open-label extension study for patients ≤ 18 years old who have participated in voxelotor clinical trials is currently enrolling participants to assess the safety and SCD-related complications of long-term treatment with this drug. Crizanlizumab is a monoclonal antibody directed against P-selectin, an adhesion factor expressed by endothelium cells which allows the formation of aggregates between platelets and leukocytes, thus contributing to vessels occlusion in the microcirculation. The SUSTAIN trial, which led to the approval of the molecule for patients ≥ 16 years old [64, 65], demonstrated a significant reduction of VOCs regardless of the concomitant use of hydroxyurea [66]. Several clinical trials on children with SCD are currently ongoing, evaluating different aspects of this new drug (Table 2). Numerous other studies are evaluating novel molecules targeting different pathophysiological mechanisms of the disease, such as inflammation, cell adhesion, and oxidative stress (Table 2). Two phase III studies are now recruiting patients ≥ 12 years old to assess the safety and efficacy of inclacumab, an inhibitor of P-selectin similar to crizanlizumab, with the difference that it can be administered every 4 months. GBT021601 is a novel HbS polymerization inhibitor, like voxelotor, which showed a higher Hb occupancy in the phase I trial [55], potentially having a greater efficacy at lower doses. A phase II/III randomized multicenter study should begin soon. Arginine hydrochloride, which is the substrate of NO production, is being evaluated in a phase III trial, including patients 3–21 years old. Complement activation is increasingly being studied as a key component of the pathogenesis of hemolysis in SCD crises [67, 68]. A phase I and a phase II trials are currently recruiting patients 15–55 years old to evaluate the safety and efficacy in preventing VOCs of crovalimab, a complement C5 inhibitor. Also, intravenous immunoglobulins target inflammation and are being studied in a phase I/II trial for patients 12–65 years old for the acute treatment of pain crises.

**Table 2:** Ongoing trial on novel pharmacological treatment under investigation in children with sickle cell disease

**Drug**	**Trial name/NCT**	**Population**	**Target**	**Phase/status**
**Voxelotor (GBT440)**	NCT02850406 (HOPE kids)	SCD patients aged 6 months to 17 years old	HbS polymerization	Phase 2, recruiting
	NCT04218084 (HOPE kids 2)	SCD patients aged 2 to 14 years old	HbS polymerization	Phase 3, recruiting
	NCT04188509	SCD patients aged 4 to 18 years old who have participated in voxelotor clinical trials	HbS polymerization	Phase 3, enrolling by invitation
	NCT03573882	SCD patients aged ≥ 12 years old who participated in the HOPE trial	HbS polymerization	Phase 3, active, not recruiting
**Crizanlizumab**	NCT03474965 (SOLACE kids)	SCD patients aged 6 months to 17 years old	Vaso-occlusion	Phase 2, recruiting
	NCT03814746 (STAND)	SCD patients aged ≥ 12 years old	Vaso-occlusion	Phase 3, active not recruiting
	NCT04657822	SCD patients aged ≥ 6 months old who have completed prior crizanluzumab studies	Vaso-occlusion	Phase 4, recruiting
	NCT04053764 (STEADFAST)	SCD patients aged ≥ 16 years old	Vaso-occlusion	Phase 2, active not recruiting
	NCT03264989	SCD patients from 16 to 70 years old	Vaso-occlusion	Phase 2, active not recruiting
	NCT03938454 (SPARTAN)	SCD patients from 16 years older	Vaso-occlusion	Phase 2, recruiting
**Inclacumab**	NCT04935879	SCD patients aged ≥ 12 years old	Vaso-occlusion	Phase 3, recruiting
**GBT021601**	NCT05431088	SCD patients 6 months to 65 years old	HbS polymerization	Phase 2/3, not yet recruiting
**Arginine Hydrochloride**	NCT04839354 (STArT)	SCD patients from 3 to 21 years old	Vaso-occlusion	Phase 3, recruiting
**Mitapivat (AG-348)**	NCT05031780	SCD patients from 16 years old	Pyruvate kinase activator	Phase 2/3, recruiting
**Crovalimab**	NCT04912869	SCD patients from 12 to 55 years old	Complement C5 inhibitor	Phase 1, recruiting
**IVIG**	NCT01757418	SCD patients from 12 to 65 years old	Inflammation	Phase 1/2, recruiting

As mentioned before, pyruvate kinase allosteric modulators have also been studied for SCD. In ex vivo treatment studies, these drugs reduced hemolysis, and sickling, and ameliorated RBC hydration through ATP-dependent channels, thus improving RBC survival [69]. Studies on SCD with mitapivat and another allosteric modifier, etavopivat (FT-4202), are ongoing [70].

In a phase I study on adults with SCD, mitapivat was safe and well tolerated and improved hemoglobin levels and hemolytic markers [71]. Preliminary results of the ongoing phase II (ESTIMATE) trial on SCD patients ≥ 16 years old confirmed the safety profile and the results of the phase I trial [72]. A phase II/III study on SCD patients ≥ 16 years old is underway [73]. Similar findings were observed with the use of etavopivat, which lead to further clinical evaluation in a phase 2/3 study [74].

Despite initial positive results with the above mentioned agent IMR-687, a phase IIb study assessing its safety and efficacy was recently terminated because it failed to meet the primary endpoint at the interim analysis [75, 76]. Several other drugs targeting different pathogenetic mechanisms of SCD have been studied, not always with positive results, but surely they all have impacted the progression of the therapeutic approaches to the disease [77–80].

## Gene therapy and gene editing for thalassemia and sickle cell disease

Allogeneic hematopoietic stem cell transplantation (HSCT) has been the only curative option for hemoglobinopathies for decades. It has provided a cure to many patients, with the best outcomes reported for young patients, in preference less than 14 years old, transplanted from a matched sibling donor [81, 82]. However, the risk of acute and chronic complications and the lack of available donors have limited its use [83].

In the last decades, new curative strategies have been developed: gene addition and gene editing, the former acting by adding a copy of a gene into the genome of the cells in the target organ or tissue, the latter by altering the DNA sequence of a gene and thus modifying its expression. Based on autologous HSCT, these strategies eliminate the need for a donor and the risks specific to allogeneic HSCT, such as graft-versus-host disease (GvHD). Different products are being studied for hemoglobinopathies (Table 3), all of which share the goal to produce exogenous β- or γ-globin chains, induce endogenous γ-globin production, or correct the SCD mutation. The aim is to restore the α/β-like globin chain imbalance in thalassemia and reduce the percentage of HbS in SCD. In June 2019, the first gene addition product, betibeglogene autotemcel (LentiGlobin BB305 [Zynteglo]), was approved by EMA for TDT patients > 12 years old who do not have a β0/β0 genotype, and for whom HSCT is appropriate, but a human leukocyte antigen (HLA)-matched related donor is not available [84]. On August 2022, the drug was approved by FDA [85]. At first, transfusion independence (TI) was obtained in adults with a non-β0/β0 genotype. The level of integration (vector copy numbers, VCN) proved to be insufficient at achieving TI for most of the β0/β0 adults, but a conspicuous reduction in transfusion burden was reached [86]. Subsequent trials managed to optimize transduction protocols, increasing the VCN and maximizing transgenic chimerism. According to the interim data as of November 2020, among the patients treated across four trials [87], 88.2% of patients showed TI maintained for a median of 20.6 months. Weighted average Hb during TI was 11.5 g/dl. There were no deaths, and no evidence of clonal dominance or insertional oncogenesis reported. Preliminary data of the long-term follow-up (LTFU) study from 44 TDT pediatric patients showed that TI was achieved and maintained in 68.2% of patients treated in the phase I-II studies and in 90.9% of patients treated in the phase III studies [87]. Serious adverse events during the LTFU study included single reports of gonadotropic insufficiency, ectopic pregnancy, fetal death, gallbladder wall thickening/polyp, bacteremia with neutropenia, and major depression, while no deaths, replication-competent lentivirus, or insertional oncogenesis were reported. The same product is under investigation in SCD patients with the name of lovotibeglogene autotemcel. In a phase I–II study all patients stopped RBC transfusions 90-day post-treatment. Complete resolution of VOCs and acute chest syndrome was observed in almost all patients. Moreover, patients reported an improvement in pain [88]. On February 2021, Bluebird Bio announced the temporary suspension of clinical trials and marketing of lovotibeglogene autotemcel following two reports of acute myeloid leukemia (AML) and myelodysplastic syndrome (MDS) later evolved in AML in SCD patients recruited in the HGB-206 trial [89, 90]. On June 2021, the company announced the lifting of FDA clinical hold for SCD and β-thalassemia studies, considering both cases to be very unlikely related to the vector [91]. These events were thus further investigated through molecular studies in order to determine if insertional oncogenesis of the pharmacological product occurred [90, 92, 93]. In both cases, this option was ruled out since the identified mutations (such as monosomy of chromosome 7) could be ascribed to the myeloablative conditioning or to the preexistent higher risk of hematological malignancy typical of all the patients affected by hemoglobinopathies. However, Bluebird Bio announced the end of commercial operations in Europe, claiming that it has been too difficult to convince European governments to pay high prices upfront for treatments that may lead to much higher savings for healthcare systems later. The company has decided to focus on the USA, where its therapies are more likely to be reimbursed at the desired prices [94].

**Table 3:** Gene therapy and gene editing ongoing trials in children with hemoglobinopathies

**Drug product**	**Disease**	**Strategy**	**Trial name/NCT**	**Population**	**Phase/status**
**Betibeglogene autotemcel (LentiGlobin)**	β-TDT	Gene therapy	NCT03207009	β-TDT patients aged ≤ 50 years old	Phase 3, active not recruiting
			NCT02151526	β-TDT patients aged 5 to 35 years old	Phase 1–2, completed (has results)
			NCT02633943	β-TDT patients aged ≤ 50 years old	LTFU of subjects with TDT treated with ex vivo gene therapy using autologous HSCT transduced with a lentiviral vector
**Lovotibeglogene autotemcel (LentiGlobin)**	SCD	Gene therapy	NCT04293185	SCD patients aged 2 to 50 years old	Phase 3, recruiting
			NCT02151526	SCD patients aged 5 to 35 years old	Phase 1–2, completed (has results)
			NCT02140554	SCD patients aged 12 to 50 years old	Phase 1–2, active, not recruiting
**OTL-300**	β-TDT	Gene therapy	NCT02453477 (TIGET-BTHAL)	β-TDT patients aged 3 to 64 years old	Phase 1–2 (status unknown, follow up study)
			NCT03275051	β-TDT patients aged ≥ 3 years old	LT safety and efficacy follow-on study in patients TDT who received OTL-300 and completed the TIGET-BTHAL study (active, not recruiting)
**DREPAGLOBE**	SCD	Gene therapy	NCT03964792 (DREPAGLOBE)	SCD patients aged 5 to 35 years old	Phase 1–2, recruiting
**BCH-BB694**	SCD	Gene therapy	NCT03282656	SCD patients aged 3 to 40 years old	Phase 1, active, not recruiting
**CTX001**	β-TDT	Gene editing	NCT03655678	β-TDT patients aged 12 to 35 years old	Phase 2–3, active not recruiting
			NCT04208529	β-TDT patients aged ≥ 2 years old	LTFU study of subjects with β-thal or SCD treated with CTX001 (enrolling by invitation)
**Gene edited autologous HSC with β-globin restoration**	β-TDT	Gene editing	NCT04205435	β-TDT patients aged 5 to 15 years old	Phase 1–2, enrolling by invitation
**Gene edited autologous HSC with γ-globin expression**	β-TDT	Gene editing	NCT04211480	β-TDT patients aged 5 to 15 years old	Phase 1–2, recruiting
**CTX001**	SCD	Gene editing	NCT03745287	SCD patients aged 12 to 35 years old	Phase 2–3, active not recruiting
			NCT04208529	SCD patients aged ≥ 2 years old	LTFU study of subjects with β-thal or SCD treated with CTX001, enrolling by invitation
**OTQ923 and HIX76**	SCD	Gene editing	NCT04443907	SCD patients aged 2 to 40 years old	Phase 1–2, recruiting

Another phase I–II trial on patients with TDT used a lentiviral vector (the GLOBE vector) to add a *β*-globin gene to autologous HSCs with intrabone administration of the transduced cells. The best outcomes were reached in the pediatric cohort, with 3 out of 4 children achieving TI [95]. The LTFU study is currently in progress, and preliminary data are awaited [96]. Also, this vector is now under evaluation in a phase I–II trial for patients with SCD [97].

Another novel gene therapy targets BCL11A, a transcription factor that represses γ-globin expression. Inhibiting BCL11A increases the expression of HbF [98]. A trial using a lentiviral vector, BCH-BB694, to introduce a short hairpin RNA (shRNA) to decrease BCL11A expression, which led to consistent and stable HbF increase, was conducted initially in 6 SCD patients with consequent clinical improvement [99]. Additional phase I/I–II trials for different lentiviral products are in progress for SCD, but preliminary data are not yet published. Other studies are based on gene editing techniques, mainly by CRISPR-Cas9 and mainly target an erythroid enhancer element of BCL11A (Table 3). The most promising is exagamglogene autotemcel (formerly CTX001), a CRISPR/Cas9-modified autologous HSCT product currently investigated in TDT and SCD. Results from the first 44 TDT patients treated with CTX001 showed significant increase of HbF, and achievement of TI in 42 patients, while 2 patients showed significantly decreased transfusion burden [100]. Similarly, 31 SCD patients achieved significantly increase of HbF and remained free of VOCs for a period of 2 up to 32.3 months [101]. Additional gene editing techniques like base editing will further advance the field and may provide more therapeutic options for hemoglobinopathies. Though gene therapy and gene editing techniques eliminate the need of donor and the risk of developing GvHD, these procedures still carry risks and challenges [102], especially concerning conditioning regimens, which increase infection and oncogenic risk. Lastly, it is worth to mention that a phase I study is underway investigating the safety of in utero HSCT in fetuses with α-thalassemia major performed at the time of in utero transfusion of RBC [103]. Transplanting maternal cells into the fetus takes advantage of existing maternal–fetal tolerance during pregnancy, allowing the engraftment of transplanted cells without conditioning [104–106].

## Conclusions

The advances of the understanding of hemoglobinopathies and the improvement of the production techniques have paved the way to the development of several novel treatment approaches and new therapies. The efficacy and safety of many new therapies in both children and adults are still under study. In the coming years, the therapeutic approach to congenital anemias is going to change and became more and more complex, aiming to change the natural history and the burden of the disease for these disorders.

## Abbreviations

2,3-DPG: 2,3-Diphosphoglycerate
ATP: Adenosine triphosphate
cGMP: Cyclic Guanosine monophosphate
EMA: European Medicine Agency
FDA: Food and Drug Administration
GvHD: Graft-versus-host disease
Hb: Hemoglobin
HSCT: Hematopoietic stem cell transplantation
LTFU: Long-term follow-up
NTDT: Non-transfusion-dependent thalassemia
PKR: Pyruvate kinase
RBC: Red blood cell
SCD: Sickle cell disease
TDT: Transfusion-dependent thalassemia
TGF-β: Transforming growth factor beta
TI: Transfusion independence
VOCs: Vaso-occlusive crises
